# Regional Issue: Social Policy Developments in Australia and New Zealand

**DOI:** 10.1111/spol.12037

**Published:** 2013-10-07

**Authors:** Christopher Deeming

**Affiliations:** Geographical Sciences, University of BristolBristol, UK

**Keywords:** Social class, Social democracy, Welfare state analysis, Australia, New Zealand

## Abstract

In his celebrated work of comparative policy, Francis Castles argued that a radical wage-earning model of welfare had evolved in Australia and New Zealand over the course of the 20th century. The Castles' thesis is shown to have two parts: first, the ‘fourth world of welfare’ argument that rests upon protection of workers; and, second, an emphasis on the path-dependent nature of social policy. It is perfectly possible to accept the second premise of the argument without the first, and indeed many do so. It is also possible to accept the importance of wage level protection concerns in Australasian social policy without accepting the complete fourth world thesis. This article explores the path of social democracy in Australia and New Zealand and the continuing importance of labour market regulation, as well as considering the extent to which that emphasis still makes Australasian social policy distinctive in the modern age. The argument focuses on the data and policies relating to labour market protection and wages, as well the systems of welfare and social protection, and the comparative information on poverty and inequality*.*

## Introduction

This article considers the path of social democracy in Australia and New Zealand, as well as the latest set of welfare reforms. We find that the individualized wage earning model of welfare laid out in the formative years of the 20th century continues to shape, if not constrain, collectivist solutions to some of the inherent social risks faced by citizens in both countries today. Underpinning the original wage earning account of welfare, as portrayed by Francis Castles, were social statistics relating to public spending from the 1950s, 60s and 70s. In this article, we also draw upon cross-national trend data from the Organisation for Economic Co-operation and Development (OECD) to help situate current developments in Australasian social policy.^1^ We are particularly interested in recent history, from the 1980s onward.

The first section of this article examines Castles' (1985) thesis on working-class welfare: *The Working Class and Welfare: Reflections on the Political Development of the Welfare State in Australia and New Zealand, 1890–1980*, before reflecting on the pace of change in social policy during the 1980s and 1990s. We then turn our attention to the distinctiveness, and thus positioning, of both Australia and New Zealand in the debates over the different worlds of welfare. In the next section, we consider the revival of social democracy in the early part of the 21st century and the new welfare reforms within the broader context of Australasian social policy, reflecting on the path-dependent nature of reform, and the extent to which the ‘Antipodean’ pathway may still be considered distinctive in the neo-liberal age. The review is particularly timely given the growing international interest in Australasian social policy in these austere times – with welfare retrenchment underway in many advanced economies. Indeed, the Australasian model of containing welfare expenditures is beginning to look much more attractive in Europe where there is widespread concern about welfare costs outstripping revenues.

## A Tale of the Workers' Welfare State, 1890–1980

Castles' basic premise was that welfare in Australasia had developed along a different pathway from that taken by European countries. Social policy emphasized the protection of working-class families, which led Castles to argue that ‘wage-earners’ welfare states' had evolved in Australia and New Zealand (Castles 1985; the original thesis was revisited and developed many times, e.g. Castles 1989, 1992, 1994, 1997a, 1997b; Castles and Uhr 2005). Social democratic efforts were directed at securing acceptable conditions of work, including legislative measures to ensure a fair minimum ‘family wage’ (consisting of a man, his wife and children). The social policy framework stood in sharp contrast to the institutional arrangements of state welfare in the Scandinavian countries of Denmark, Norway and Sweden (Castles 1978; Korpi 1978; Castles 1988). There redistributive social policies from the 1930s were designed to promote equality as a citizen's right. In Australasia, by contrast, redistributive efforts were achieved using the instruments of wage regulation rather than traditional tax-and-spend welfare policies, as seen in the Scandinavian countries.

The basis of the claim for a formative pathway in Australasian social policy relates to decisions taken at the turn of the 19th century to introduce systems of industrial regulation. The 1890s were difficult times and a time of class struggle, workforce unionization and the creation of the Labor Party in Australia (in 1891, Australian spelling, ‘Labor’, generally known as the ALP or Labor) and parties of the left in New Zealand, for example the Socialist Party (1901) and Labour Party (1910/1916). Minimum wage laws were introduced in South Australia (1894) and New Zealand (1894) and a basic wage judgement in Australia soon followed: the ‘Harvester Judgement’ (1907) (Herscovitch and Stanton 2008). These acts gave courts the power to determine (minimum) wages and working conditions. The Harvester criterion, based on the breadwinner-homemaker model, provided the grounds for social justice: minimum wages had to be sufficient for a man to provide for his family. As the distinctive focus of Australasian social policy centred on wage regulation, Castles' pursued the idea of the wage-earning model of welfare. His argument points to class, and working-class party politics in particular, as a crucial factor influencing welfare policies in Australasia; others, however, saw labour in a much broader political constellation (e.g. Watts 1997). Nevertheless, trade unionists and social reformers in Australia and New Zealand had succeeded in their fight for just wage prescriptions defined by the state. The Labour Party in New Zealand and Labor in Australia pursued other forms of occupational welfare for dealing with absence from employment due to sickness, disability or retirement from work in later life (Castles 1992).^2^ Economic policy followed Keynesian principles in both countries. The value of investment was moderated by the government to maintain the economy in a full state of employment, a tenet of Labour policy from 1936 in New Zealand and Labor policy from 1945 in Australia.

There was little political appetite for social insurance or an expanded system of welfare paid for out of general taxation because wage control was the means for securing needs-based welfare, or welfare ‘by other means’ (Castles 1989).^3^ Accordingly, in the ‘New World’ most workers owned their own homes and were able to maintain a decent standard of living for their families; thus, high home ownership rates helped sustain the wage earners' model (Murphy 2000; Dalton 2010).^4^ Also in retirement, mortgage-free homes helped to reduce pressures on the public finances. Home ownership thus acted as a form of insurance in lieu of public welfare (Castles 1998). For anyone outside the labour market, however, the standard of living was markedly different – and the thrust towards self-reliance weighed unevenly across different groups in society (Thomson 1998). Indigenous families, sole parents, older people and people with a disability were particularly vulnerable to poverty, reliant on meagre, fragmentary welfare services or more often on private charity. In New Zealand, for instance, the Widows Benefit (1911) assisted widows with children and the introduction of family assistance (the world's first in 1926) helped families with three or more children. Assistance was paid to mothers as a supplement to male wages; single mothers often found they were not eligible, however, as (absent) fathers were required to sign the benefit application. The state recognized the family as the unit not the individual and benefits were paid to support this unit. The rate of family allowance in New Zealand increased under the Social Security Act (1938), although universal family benefit was not achieved until 1946 (Nolan 87). In Australia, the Liberals objected to the ‘family wage’ policy and attempted to undermine it (Bessant *et al*. 2006). They believed the pay floor distorted the labour market, and perhaps just as importantly, they saw a moral hazard: the wage policy clearly favoured single men without family responsibilities (Garton and McCallum 1996). In an attempt to restrain wages, child benefits were introduced in 1941. With the introduction of widow's pensions in 1942 and sickness and unemployment benefits in 1945, the Australian ‘welfare state’ was created (Herscovitch and Stanton 2008).

**Figure 1:**
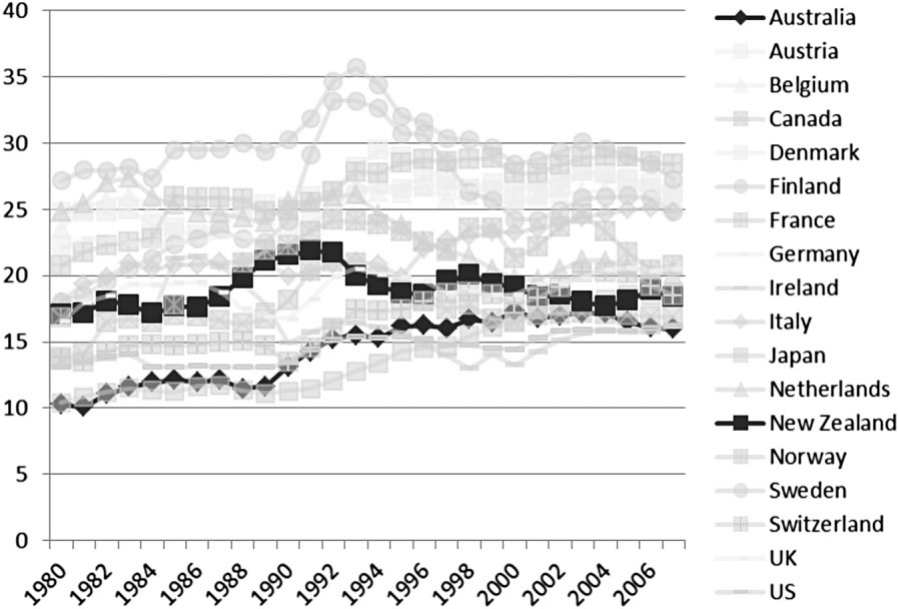
Public expenditure on social welfare in OECD countries (gross expenditure as a % of GDP), 1980–2012 *Source:* OECD Social Expenditure Database (SOCX), http://stats.oecd.org (accessed April 2013).

The tale of the workers' welfare state in the ‘Antipodes’ was supported by early comparative welfare statistics, which begin to emerge with the founding of the OECD (established in 1960) in Paris (OECD 1972). We find public spending on social welfare in Australia and New Zealand was low compared to the other advanced economies. During the 1950s and 60s, total expenditure on social security was around 5 per cent of gross domestic product (GDP) per annum in Australia and about 6 per cent in New Zealand. The OECD average was higher at 8 per cent. The relative picture had hardly changed in the subsequent decades of the 1970s and 80s. In Australia, the total welfare bill averaged 13 per cent of GDP each year during the 1970s, compared with 15 per cent in New Zealand and an OECD average of 19 per cent. By the mid-1980s, when Castles' published *The Working Class and Welfare*, welfare spending in Australia trailed the OECD average by eight percentage points with a lag of three percentage points in New Zealand. Only Japan was spending less than Australia on welfare services (figure 1). Although Australia and New Zealand appeared as the conspicuous welfare laggards of the advanced world, at least in the conventional sense, Castles' work challenged the view that high public spending would necessarily indicate a better system of welfare in the ‘Antipodes’. Another interpretation of high welfare spending in this context might equally signal that family and market structures were failing to cope (Castles 1992). In the ‘Antipodean’ context, the instruments of ‘pre-distribution’ – the way in which the market distributes its rewards, before government gets involved – were the means to achieve a more equitable distribution of market incomes.

## Caught in the Middle: Neo-liberal Reforms in the 1980s and 1990s

As with many other OECD countries during the 1980s, both Australia and New Zealand pursued neo-liberal policies or ‘economic rationalism’ based on ideas about the supremacy of free markets and the efficiency of market forces. In the ‘Antipodean’ context, however, New Right ideology assumed particular significance, with state intervention and protection in the economy now widely accepted to be distorting the functioning of the market. The wage-earning model of welfare was in need of ‘refurbishment’ in order to cope with the issues of the day (Castles 1994).

**Figure 2:**
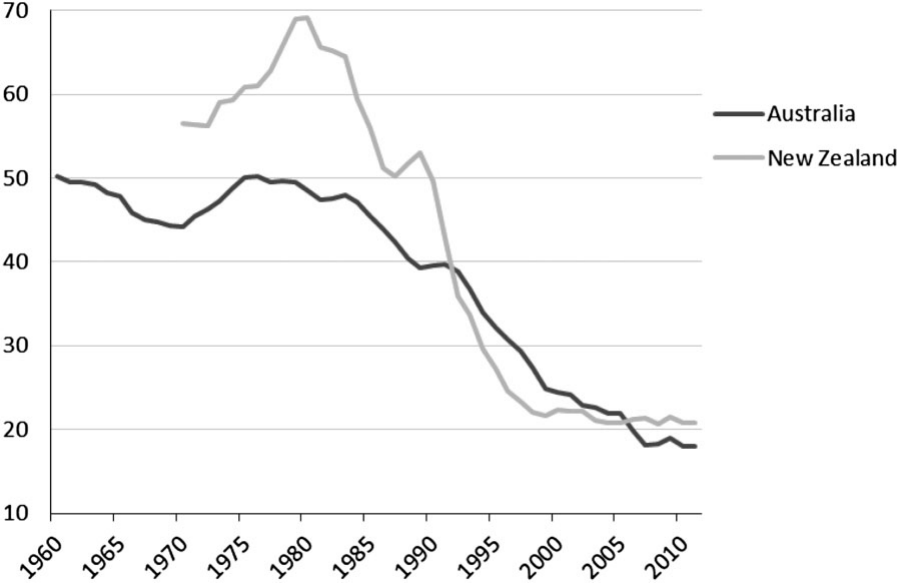
Trade union membership in Australia and New Zealand (% of all employees), 1960–2011^α^ *Source:* OECD Database on Trade Unions, 1960–2011, http://stats.oecd.org (accessed April 2013). *Note:* α = trade union density corresponds to the ratio of wage and salary earners who are trade union members, divided by the total number of wage and salary earners.

Although the two countries followed a similar path towards free-market reforms, the Australian Labor government (elected in 1983) was much more cautious than its New Zealand counterpart (elected in 1984).^5^ It claimed to be navigating a ‘middle way’ between old-style or ‘classical’ social democracy and neo-liberal adherence to the free market. The Australian Labor government, for example, (re-)introduced Medicare, the national health insurance scheme that provided universal access to hospital and medical services.^6^ In New Zealand, by contrast, the Labour government had few, if any, significant achievements in the field of social welfare. The divergence between the two countries was particularly evident in the area of labour-market policy (Barry and Wailes 2004). New Zealand's fourth Labour government embarked upon a more radical labour-market reform aimed at removing the influence of the unions in the pay bargaining process. The Labour Relations Act (1987), for example, liberalized the labour market and the State Sector Act (1988) extended these reforms to the public sector. In Australia, the Accord (1983) between Labor and the Australian Council of Trade Unions (ACTU) ushered in a new era for industrial relations policy and a centralized system of wage fixation.^7^ It was through the Accord, for example, that the Keating Labor government introduced a compulsory company-based superannuation scheme in 1992. Under the Superannuation Guarantee Charge (SGC) employers were required to set aside contributions for their employees into privately run superannuation funds. Labour market policy converges again a decade later; the Industrial Relations Act (1993) (under Liberal auspices) in Australia freed the labour market, thus aligning Australian industrial relations policy more closely with the New Zealand model. Unemployment was accepted by Labor as a necessary cost of reforming the economy (Quiggin 1998). Trade unions were marginalized and workers increasingly lost faith in them, as evidenced by the dramatic decline in trade union membership over this period (see figure 2).

During the 1980s, social security also became more ‘targeted’ and ‘selective’ (Saunders 1991; Stephens 1999). Concerns about ‘welfare dependency’ led to acceptance of ‘active’ welfare and labour market policies. Work offered a way to escape poverty through more generous in-work wage supplementation. Tax credits were introduced to help ‘make work pay’. The Family Support tax credit (1986) in New Zealand, for instance, provided targeted assistance to low income families in the form of tax concessions. By the 1990s, ‘economic rationalism’ was more widely accepted in the ‘Antipodes’ and the National and Liberal governments pursued ever more radical free-market reforms (Saunders 1994; Boston *et al*. 1999).

In Australia, the Liberal government, elected in 1996, turned away from universality in the social services, promoting private-sector expansion instead. It attempted to drive a clearer divide between the public/private sectors in areas of social welfare, often through tax concessions, such as the Private Health Insurance Rebate introduced in 1996. Tax changes were dominated by the introduction of the Goods and Services Tax (GST), a tax on consumption, in 2000, some 14 years after GST was introduced in New Zealand. In the pursuit of market-orientated reforms, the Accord with the unions was abandoned. The Workplace Relations Act (1996), for example, completely undermined the long-established system of independent arbitration. The Liberals were looking to increase national economic performance, and, following standard neo-liberal philosophy, they firmly believed that a more flexible and active labour market was necessary to achieve this. The Workplace Relations Amendment Act 2005, popularly known as WorkChoices, saw further deregulation of the wage structure and the removal of employment laws relating to unfair dismissals. Critics maintained that the laws stripped away the basic rights of employees and were fundamentally unfair. Certainly, the principal thrust of the WorkChoices legislation was to individualize employment relations, with the effect of marginalizing trade unions and industrial tribunals.

Financial and labour market deregulation was more rapid in New Zealand, with deep cuts in government spending in the face of recession and high levels of unemployment. The centre-right National Party in New Zealand, which had won the 1990 election, pursued harsh austerity measures in what has been called the ‘mother of all budgets’, arguably the most dramatic example of welfare state retrenchment not only in New Zealand, but in the OECD as a whole (Starke 2008). Family Benefit was abolished in 1991, welfare benefits were drastically cut (by up to 25 per cent), and the state pension age was raised. As in Australia, further changes to eligibility criteria increased the targeting of social security benefits in order to constrain government expenditure (Saunders 1999). Means-tested assistance focused only on those deemed to be most in need. The reforms in New Zealand, however, had begun much earlier than in Australia and were much more radical. Arbitration was completely abolished under the Employment Contracts Act (1991), giving unions no rights of any kind. Public services underwent reform as the ‘quasi-market’ economy replaced government bureaucracies in the pursuit of efficiency and user-charges were introduced for many public services that were formerly free (Ashton *et al*. 2005). Unsurprisingly, perhaps, these policies had the desired effect of reducing New Zealand's national debt and were therefore judged a success. Certainly, at this time, the radical approach to reform in New Zealand was widely regarded as the correct or standard model of neo-liberal policy, yet Australia outperformed New Zealand on a range of important social and economic indicators (Castles *et al*. 1996).

Social policy in Australasia for much of the 20th century had been geared towards ensuring a fair working wage; male workforce participation was the primary way that family welfare was secured. Social citizenship entitlements were weak vis-à-vis the rights of wage earners and non-wage earners. The state in both countries was evidently failing to provide adequate social protection from poverty and low living standards for all citizens, especially non-wage earners outside the labour market without an adequate income floor (Veit-Wilson 1998). In the absence of a human right to an adequate income, it may not be surprising to find the public commitment to social welfare in the ‘Antipodean’ context trailing other OECD countries at the close of the last century (figure 1). Surveys at the time suggested that the industrial relations reforms were unpopular with the Australian and New Zealand electorate; trade unions had been a key institution of civil society, as was the protection of workers by industrial legislation (Van Wanrooy 2007; Humpage 2008).

What all this adds up to in comparative social policy terms has been the subject of controversy, as Murphy (2006) observes, with much of the dispute focusing on the work-welfare nexus in modern economies. For some observers, the architecture of the Australasian welfare state was characteristically liberal in design, recognizable by the residual system of social security based on means-tested social assistance schemes and targeted income-support benefits. Certainly, Esping-Andersen (1990) in his original work, *Three Worlds of Welfare Capitalism*, argued that Australia and New Zealand belonged to the liberal world of welfare capitalism, along with the other Protestant Anglo-Saxon countries, such as the UK, the USA, and Canada (table 1). By contrast, Francis Castles provided one of the earliest challenges, at least in part, to Esping-Andersen's welfare typology. According to Castles and Mitchell (1990, 1993), Australia and New Zealand were ‘other worldly’, being significant social policy innovators (along with the UK). They belonged to a *fourth* ‘Radical’ category of welfare capitalism within the alternative ‘families of nations’ classification posited by Castles and Mitchell, as depicted in table 1. Accordingly, government expenditure levels and the progressive income tax helped to provide the justification, along with the regulation of the labour market and the early minimum-wage laws. This positioning of Australasian social policy has attracted followers, who also agree that the Australian and New Zealand model of welfare cannot easily be accommodated within the original three-worlds typology presented by Esping-Andersen (e.g. Korpi and Palme 1998; Huber and Stephens 2001; Shalev 2007). Those claiming a ‘fourth world’ of welfare draw a firm distinction between *targeted* income-protection schemes found in Australasia and the more *encompassing* world of welfare found in the Scandinavian countries. Both of these may be distinguished from the *basic* or residual model of welfare services found in the Anglo-Saxon countries and the *segmented* systems, based on labour-market position, found in conservative countries such as France and Germany. Interestingly, Esping-Andersen was later persuaded by the case for exceptionalism in Australasia (Esping-Andersen 1997), while Castles (2001) went on to argue that the Australasian countries were being drawn closer to the ideals of the liberal world. Perhaps erroneously with hindsight, as we shall see in the next section, Castles claimed that Labo(u)r had abandoned its commitment to wage-earners' at a time when the effects of labour market deregulation began to bite.

**Table 1:** Worlds of welfare capitalismα

	Liberal	Conservative	Social-Democratic	Radical
Esping-Andersen (1990)β	Australia Canada Ireland New Zealand UK USA (Switzerland)	Finland France Germany Italy Japan (Switzerland)	Austria Belgium Denmark Netherlands Norway Sweden	
Castles and Mitchell (1990, 1993)γ	Ireland Japan Switzerland USA	Germany Italy Netherlands	Belgium Denmark Norway Sweden	Australia New Zealand UK

*Notes*:  α = welfare regimes considered to be prototypical are shown underlined.

β= Switzerland was cross-classified, being Conservative on Esping-Andersen's combined index of decommodification (Esping-Andersen 1990: 52) and Liberal on his social stratification index (Esping-Andersen 1990: 74).

γ = the four countries of Austria, Canada, Finland and France were not accommodated within Castles' and Mitchell's classification.

## Social Democracy for the 21st Century

**Figure 3:**
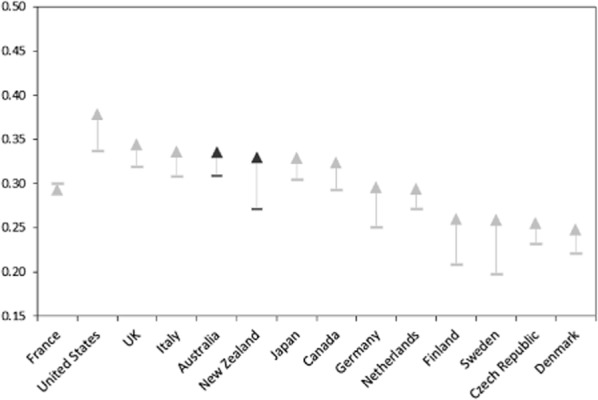
Income inequality in OECD countries, mid-1980s and late 2000s^α^ *Source:* OECD 2011a. *Note:* α = Gini coefficients of income inequality; arrows indicate the change and direction of income inequality between the mid-1980s and late 2000s.

The political and economic fortunes of Australia and New Zealand continue to display some remarkable parallels at the start of the 21st century. We find social democracy was revived in both countries, with Labo(u)r administrations attempting to chart a distinctive course in the face of dominant ‘neoliberal’ ideology (Dean 2012). The priority for both Labor in Australia and Labour in New Zealand has been to strengthen the system of welfare for ‘working families’. The notion of ‘hard-working families’ (e.g. Gillard 2010) is not simply a rhetorical device aimed at a political constituency – those on low and middle incomes – but articulates a range of normative assumptions about the way in which life should be lived in 21st century capitalist society. Work is seen as the best form of welfare, not only because work pays better than welfare but also because it promotes participation, inclusion and well-being. Therefore, one of the key goals of Australasian social policy has been to help people move from welfare benefits and into paid work. Benefit claimants in both countries discovered new conditions and new forms of enforcement attached to social security payments: being available for work and actively seeking employment, and being able to demonstrate this, for example, were minimum requirements. The role of the state was to facilitate ‘activation’, with new training programmes to improve skills and increase job readiness. This is because the goal of ‘workfare’, or ‘welfare-to-work’ as it is usually known, is not simply to reduce unemployment but also to also tackle the wider problem of worklessness. Long-term unemployment can deskill people. Workers, with specialized skills, and whose lives had been devoted to production required training that would allow them to find new work in the post-industrial service economy. Training unemployed people in the skills required by employers is therefore part of the supply-side approach adopted by the Labor governments in Australia and Labour in New Zealand to overcome shortfalls in the economy. As we shall see, a longstanding concern of social policy scholars in Australasia has centred on the welfare of those not in paid work. These are concerns now heightened by the context of rising inequality in both countries seen since the publication of *The Working Class and Welfare* in 1985 (figure 3). The gap between out-of-work benefits and in-work income is increasing in New Zealand, exacerbated by more generous wage supplementation – usually in the form of working tax credits – and reductions in the real value of out-of-work benefits.

The fifth Labour-led government of New Zealand came to power in 1999 under Helen Clark. The government moved quickly to reform the labour market. The controversial Employment Contracts Act was repealed and replaced by the Employment Relations Act (2000), which re-regulated the labour market after a decade of market liberalization. With New Zealand recovering from the recession of 1998–99, the government was committed to maintaining a tight grip on state spending. Increasing labour market participation was, therefore, the main priority; spending on social welfare was not (Clark 2002). Thus, employment and labour market activation were central to the direction of social security reform. The Working for Families (2005) welfare package, for example, was designed to help low-income households. Family Tax Credits ensured recipients were better off financially in work than they would have been on out-of-work benefits.

Social democratic ideas about ‘new welfare’ and ‘social development’ now defined social policy (Craig 2006; Lunt *et al*. 2008). New activation schemes were introduced to support people ‘into work’. Intensive employment services and training programmes helped to ensure a highly skilled workforce and a high level of labour market participation. At the same time, the benefits system was simplified. ‘Core benefits’ such as the Minimum Family Tax Credit replaced existing ones like the Family Support and the Guaranteed Minimum Family Income. Income adequacy and poverty prevention for those dependent on social security benefits provided by the state were, however, accorded a much lower priority (Christine *et al*. 2009). After the 2008 and 2011 general elections, the conservative National Party formed a government. The radical neo-liberal programme of welfare reform was backtracked under Labour, but only to a degree (Cheyne *et al*. 81). The fiscal market changes have endured, as have the competitive and quasi-market approach to organizing and delivering public services (Tenbensel *et al*. 2011), and employment relations reforms in the direction of labour-market flexibility are anticipated under National (Haworth 83).

In Australia, the global financial crises helped to usher in a new era of social democracy (Saunders and Deeming 2011). The Rudd Labor government came to power in 2007. In the face of recession, the Rudd government, like others around the world, turned to Keynesian policies to stimulate the economy. Labor set itself apart from New Right ideology, arguing instead for social democratic values and principles of ‘social investment’ and inclusive economic growth (Smyth and Buchanan 2013). As in New Zealand, the Labour government moved to strengthen the social policy framework for ‘working families’; with positive connotations, this term was extensively used by Kevin Rudd and Julia Gillard (Australian Prime Minister 2010–13) during the 2007 federal election. Successive Labor budgets over the last four years (2008–12) have been mildly progressive, intent on improving living standards for ‘hard-working families’ (Australian Government 2008: 4). The Fair Work Act (2009) reversed the unpopular labour market polices of the 1990s and provided workers with a safety net of employment conditions. In particular, Labor restored to Australian workers the legal right to appeal against harsh or unfair dismissals from their place of work – a right that had been rescinded by WorkChoices. Once again, collective bargaining is encouraged and wages are to reflect relative living standards and the needs of the low paid (with due consideration of the potential impact of changes on the labour market and unemployment levels). Like its international counterparts, the ALP is now content to pursue the (diminished) goal of full employability (as opposed to full employment), with mutual obligation and increased investment in training provision being key to helping unemployed people to return to the labour force. Under Labor, there have been modest reforms to the welfare system, particularly in the areas of aged pensions, healthcare, paid maternity leave and Labor's social inclusion agenda, discussed by Saunders (2013) in this issue. Recent reform discussion has focused on better integration of the tax and benefit arrangements in the superannuation system. The present Labor administration is proposing further extension of the superannuation scheme to 12 per cent of wages, which, whatever its deficiencies, will deliver much better outcomes for working people (and the middle-class) than the British system. It has also introduced the National Disability Insurance Scheme (NDIS) and Disability Care Australia to support the 460,000 Australians who have a significant and permanent disability. However, the system of targeted social security for the poorest members of society remains intact. The government resisted calls to reform the system of family benefits recommended by the Henry Review (2009). While more fundamental reforms to the tax and transfer system are unlikely, the Henry Review concluded that the present system, which has long favoured market freedom and individual opportunity, had served Australia well. Action was taken to raise the basic pension following the Harmer Review (2009) of pensions: the minimum income floor for pensioners now stands at 28 per cent of average weekly male earnings. Another key priority for Labor has been to strengthen the Australian national health service, which has long been fragmented on state lines and among the different tiers of government. Reforms to the funding, delivery and organization of health services require the commonwealth, state and territory governments to work together to improve healthcare services (National Health and Hospitals Network Act 2011).

**Figure 4:**
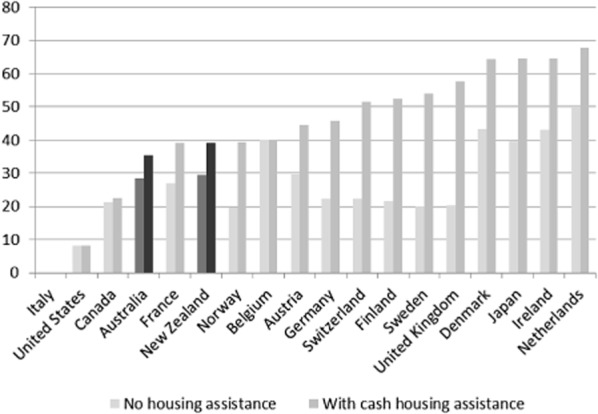
Income levels provided by minimum-income benefits for adults in OECD countries, 2010^α^ *Source:* OECD Income Distribution Database, www.oecd.org/els/social/inequality (accessed April 2013). *Note:* α = net income value in % of national median equivalized household incomes.

While Australasian social democratic reforms show strong continuities with the neo-liberal agenda, there are also critical differences, particularly with the role of the ‘enabling’ or ‘social investment’ state in Australia and ‘social development’ state in New Zealand taking greater responsibility for workforce participation. The traditional social democratic concerns with social justice and social investment remain, but the relationship between the state and recipients of welfare has been recast in a new contract of ‘reciprocal obligation’, of ‘rights and responsibilities’. Social policy scholars continue to question the adequacy and effectiveness of the ‘Antipodean’ social security framework (e.g. O'Brien 2008; Saunders 2011; Soldatic and Pini 2012). According to the OECD, welfare safety nets and out-of-work benefits are not wholly adequate for escaping poverty or meeting the needs of the most vulnerable members of society (OECD 2011a). Minimum-income benefit levels in Australia and New Zealand are well below the international poverty line of 60 per cent median income. Calculations are shown in figure 4 (showing housing benefits included and excluded).

**Figure 5:**
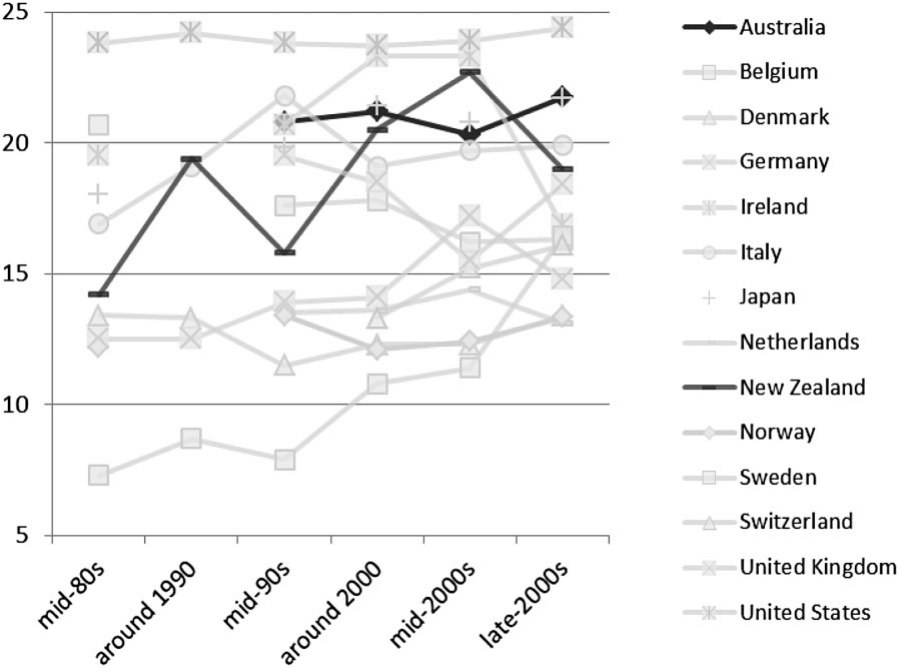
Poverty rates in OECD countries, 1985–2010^α^ *Source:* OECD Income Distribution and Poverty, http://stats.oecd.org (accessed April 2013). *Note:* α = poverty is defined at 60% of national median equivalized household income after taxes and transfers.

Income poverty levels are relatively high in Australia and New Zealand, compared to most other advanced economies (figure 5). The risk of poverty is not distributed evenly within society. People who are unemployed and jobless, sole parents, adults with a disability, and Indigenous people face a higher risk of poverty. Saunders (2011), for instance, argues that it would require only modest increases in social security payments to improve the standing of Australia in the international poverty league table, but work incentives have long taken priority over adequacy in Australasian social protection policy (Stephens 2000; Deeming 86; O'Sullivan and Ashton 85).

**Figure 6:**
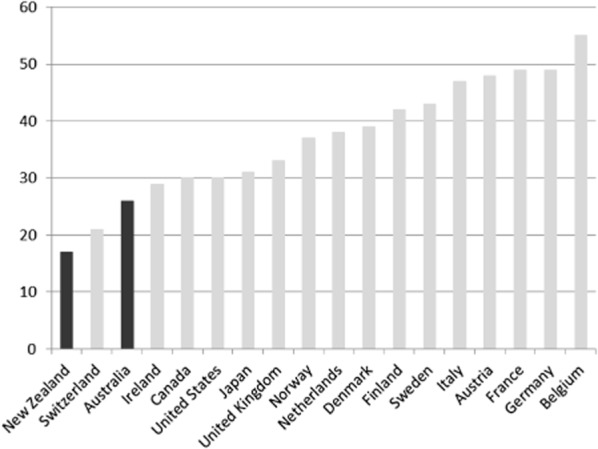
The tax burden on waged income in OECD countries, 2010^α^ *Source:* OECD Tax, http://www.oecd.org/tax/ (accessed April 2013). *Note:* α = average personal income tax and social security contribution rates for adults at 100% of average wages.

The Australasian tax-transfer systems play ‘Robin Hood’ and target assistance to poor people in two ways. First, through means-testing, with very high means-test thresholds, in order that payments to better off households are minimized (Immervoll 2010). By international standards, the Australian tax and benefits systems are progressive (OECD 2008) and the system of cash transfers and income redistribution remains one of the most targeted (and progressive) in the Western world (OECD 2010). Second, targeting is achieved through low levels of direct taxes on low-income households, to ensure that very little of the assistance directed to lower income families is clawed back. Thus, the current tax burden on waged labour, shown in figure 6, is low by the standards of most developed economies.

**Figure 7:**
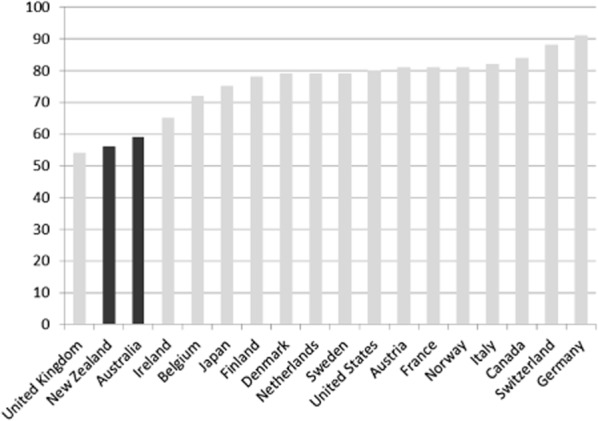
Unemployment benefit replacement rates for families in OECD countries (based on average wages in 2005) *Source:* OECD 2007.

As Australasians experience levels of decommodification that are amongst the lowest in the Developed world, they accept the risk of a relatively low standard of living if they are unable to work or become unemployed (OECD 2007). Benefit replacement rates, paid in the initial phase of unemployment, for example, amount to about one-third of average wages in both Australia and New Zealand; only unemployed adults in Ireland receive less. The situation for families, as shown in figure 7, is not much better. Benefit replacement rates for a family with two children are below 60 per cent of average wages; only unemployed families in the UK appear worse off. Relatively low means-tested minimum-income safety nets, seen in figure 4, and low unemployment benefit replacement rates are evidently linked to the modest welfare commitment observed in figure 1.

Gender and Indigenous inequalities persist, particularly in the labour market, and continue to pose major challenges for social policy. Feminist work has shown how social policies in the ‘Antipodes’ continue to be imbued with the breadwinner ideology (O'Connor *et al*. 1999; Mitchell 2004). As such, decommodification has been critiqued as being inherently male; women (and youth) continue to be marginalized by an employment regime that tends to revolve around the needs of predominantly male workers.

**Figure 8:**
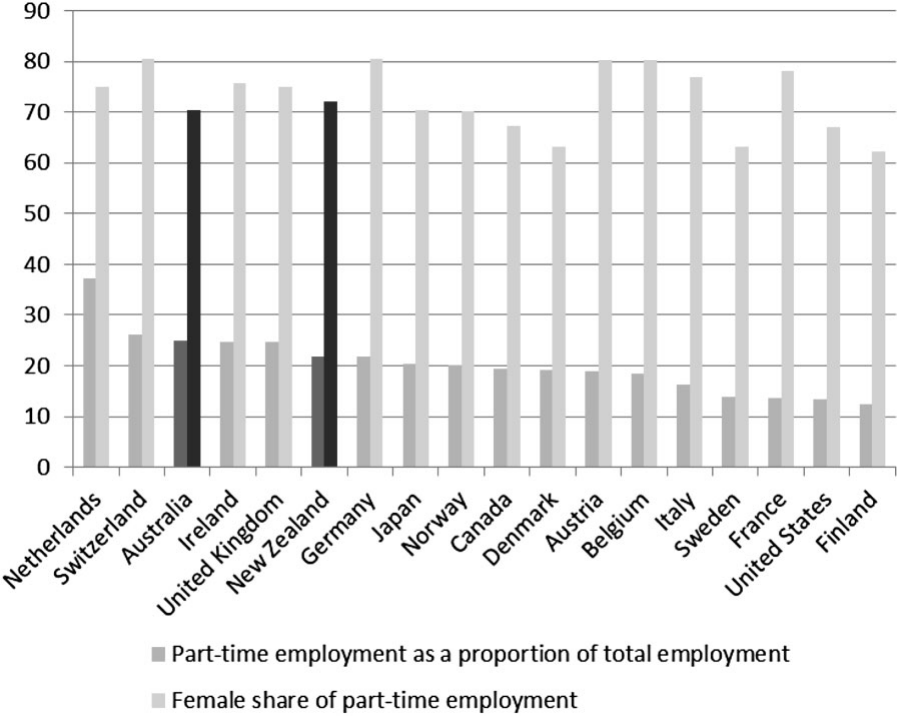
Part-time employment as a proportion of total employment, and female share of part-time employment in OECD countries, 2010 *Source:* OECD employment and labour markets, http://www.oecd-ilibrary.org (accessed April 2013).

**Figure 9:**
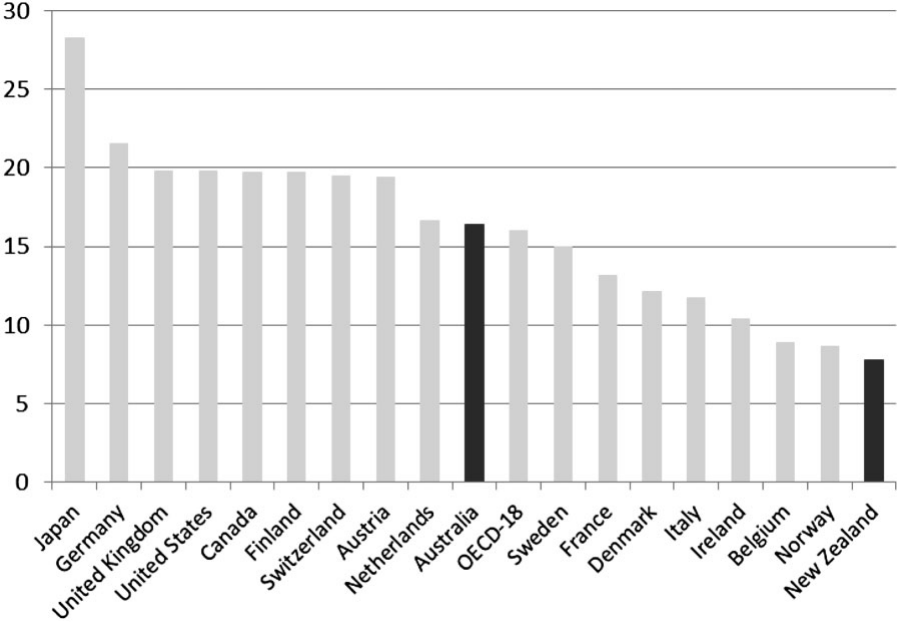
Gender wage gap in OECD countries (median earnings of full-time employees), 2009 *Source:* OECD 2011b.

Labour market ‘flexibility’ is now part of global capitalism and is the result of economic policies implemented in most countries over the last three decades. Australia and New Zealand are not exceptional in this regard. Both have very ‘flexible’ labour markets, characterized by high rates of part-time employment and casual work. However, we find that, as women are much more ‘flexible’ workers than their male counterparts, they disproportionately occupy more precarious part-time positions (figure 8). In Australia and New Zealand, the female share of part-time employment is close to 75 per cent. This level of part-time work also helps to explain the inferior labour market position of women, who take on disproportionate responsibility for care work (Lewis and Plomien 2009). We find the gender wage pay gap in Australia to be above the OECD (2011b) average, although New Zealand performs much better on this measure of equity (figure 9). Progress on tackling Indigenous disadvantage in Australasia remains very slow despite recent efforts to combat this issue such as the ‘Closing the Gap’ initiative in Australia (Humpage 2005; Australian Government 2009; AIHW/AIFS 2012). The unemployment rate for people of Aboriginal and Torres Strait Islander origin in Australia, at 20 per cent, is over three times the national average. For people from Māori and Pacific Island ethnic communities in New Zealand, the unemployment rate in the year to March 2012 was 13 per cent, compared to 6.6 per cent in the general population (Department of Labour 2012). The re-engineering of welfare policy by the latest Labor governments in Australia and Labour in New Zealand has struggled to tackle the deeply entrenched gender and ethnic inequalities, the persistent poverty and rise in inequality from 1985 (figure 3).

In sum, we find relatively low tax and social welfare spending, targeted tax cuts for ‘working families’, renewed labour market protection and the notion of appropriate minimum wage rates on social justice grounds could – if we accept the idea of pathways in social policy as Castles (2010) suggests – be seen to mark a return to more familiar territory for Australasian social policy in the 21st century. As a result of the social and economic transformations of the 1980s and 90s however, there has been a fundamental shift in the model of household welfare and production, from the male-breadwinner female-homemaker model, i.e. the ‘wage-earners' welfare state’, to a dual-income model of household welfare and production (Watts 1993). Accordingly, policy is increasingly formulated to meet the needs of ‘hard-working families’. In the discussion, we consider this proposition and offer final reflections on the distinctiveness of Australasian social policy in the early part of the 21st century.

## Discussion

The historic trade-off between ‘capital’ and ‘labour’ in the industrialized world was, arguably, the ‘welfare state’. In Australia, however, democratic welfare state building emerged from the bottom up (Castles *et al*. 2012). Thus, the distinctive focus of social policy for the protection of working-class families Australasian-style was via wage regulation. As Castles argued:

The historic compromise between the classes did not centre around a modification of the reward structure of capitalism through the distributive mechanisms at the command of the state, as in the European countries, but focused directly on the primary distribution of income generated by the capitalist market mechanism. (Castles 1985: 87)

*The Working Class and Welfare* helped to challenge the predominant view that Australia and New Zealand were welfare laggards. Castles argued that a focus on social programmes alone in the ‘Antipodean’ context does not necessarily reflect the extent to which citizens in the two nations are protected against market risks. This line of argument was not completely original in social policy, as Richard Titmuss had long emphasised the need to consider a range of welfare mechanisms within society, including the role of occupational welfare (which Castles drew attention to). For much of the last century, occupational welfare and the system of wage determination played a central role in enhancing the degree of welfare enjoyed by workers in Australia and New Zealand, and so it is timely in this Special Edition of *Social Policy & Administration* to return to the issue of distinctiveness in Australasian social policy.

First, we continue to observe the importance of democratic class politics in determining social policy outcomes in Australasia. The old male breadwinner model of welfare grew out of class struggle, and in turn, it is class politics that continue to shape, if not constrain, today's welfare settlement. The very political expression ‘hard-working families’ embodies not only a rhetorical device aimed at a political constituency, families on low-to-middle incomes; it also articulates a range of normative assumptions about life and, more importantly, work in 21st century capitalist society. In the battle over the political middle ground, middle-class families have been brought into the social security system, through the mechanisms of tax rebates and credits, which is why some observers now claim that a ‘dual welfare state’ function exists in Australia (Stebbing and Spies-Butcher 2010). The Low Income Tax Offset (LITO) in Australia is a tax rebate for those on lower incomes, and the Child Care Rebate and Family Tax Benefit (FTB) have also been extended to help meet the needs of more affluent hard-working families (Australian Government 2011); while in New Zealand the Minimum Family Tax Credit and In-work Tax Credit aid low and middle income families. Australasia is not alone in the pursuit of working tax credits; many other countries such as the UK and the USA have earned income tax credit systems and operate wage supplements. In the USA and the UK the battle is also on for the hearts and minds of ‘the strivers’, ‘the squeezed middle’ (Parker 2013). The British Labour government was very much on the side of ‘hard-working families’, i.e. families on low-to-middle incomes, during its term in office (1997–2010) and the Labour Party remains committed to the cause. The current Conservative–Liberal Democrat Coalition government is also appealing to this constituency who want to work hard and get on (HM Treasury 2012, 2013). The political appeals have become increasingly fraught as debates over rising levels of social insecurity and fairness in social policy rage, heightened by the politics of austerity.

Second, our analysis of the welfare trend data reveals important continuities in Australasian social policy. Protection against social risks, unemployment, sickness and, until recently perhaps, disability provision, remains very much an individual responsibility (the NDIS policy has the potential to revolutionize disability provision in Australia). Thus, public expenditure on social welfare is contained at relatively low levels. Ideas of welfare-state convergence are attracting support in social policy. Going forward, in the new Age of Austerity, one scenario might see Australasian distinctiveness diminish as the other advanced economies move further towards the privatization and financialization of risk in society. At a time of fiscal crises and welfare retrenchment, the Australasian model of containing the size of the welfare state is beginning to look much more attractive to outsiders (Immervoll 2010). In Europe, there is widespread concern that governments are spending too much on their welfare programmes (Adema *et al*. 2011). In the UK, the Coalition government is apparently drawing its lessons from the New Zealand experience of radical welfare reform as it attempts to embed a radical and divisive liberalism permanently in British public life (Taylor-Gooby 2013). Certainly the Coalition government's programme of restructuring across all areas of state activity and the attack on working-age welfare in an effort to cut public spending has been profound, from healthcare reform to the introduction of the new simplified ‘Universal Credit’ means-tested benefit. Beyond austerity then (IMF 2012), the ‘Australasian way’ of providing welfare is likely to look much less distinct from a cross-national comparative social policy perspective.

## Notes

OECD data is generally regarded as being robust for drawing international comparisons on economic statistics (Giovannini 2008).

New Zealand was a pioneer of social protection with the Workers Compensation Act (1897), the Old Age Pensions Act (1898) and Widows Benefit (1911). In Australia, the New South Wales Old-Age Pensions Act (1900), Victoria (1901) and Queensland (1907) followed suit; followed by the Commonwealth Age Pension Act (1908) and the Disability Pension (1909).

The Lyons' government in Australia tried to introduce a comprehensive welfare insurance scheme in 1938 – covering health and pensions – but the scheme was not implemented. Contributory insurance was opposed by Labor, as well as other vested interests including doctors and charities, as it did not cover unemployment benefits to be funded from consolidated tax revenue.

Australia, for instance, had a home ownership rate near 50 per cent in the early 1900s, reaching 70 per cent by 1960 (Dalton 2010).

New Zealand has a unitary government with a unicameral Parliament, while Australia has a federal system and bicameral national Parliament. This helps toexplain, at least in part, why policy change can be both faster in New Zealand and more extreme.

Medibank had been dismantled by the Liberal government in 1978.

The Prices and Incomes Accord on Economic and Social Policy between ACTU and the ALP ushered in a new era of industrial relations with the election of the Hawke Labor government in February 1983. Unions agreed to modest growth in real wages and the Labor government pledged to minimize inflation.

## References

[b1] Adema W, Fron P, Ladaique M (2011). Is the European welfare state really more expensive?.

[b2] Ashton T, Mays N, Devlin N (2005). Continuity through change: The rhetoric and reality of health reform in New Zealand. Social Science & Medicine.

[b3] Australian Government (2009). Closing the Gap On Indigenous Disadvantage: The Challenge For Australia.

[b4] Australian Government (2011). Budget Overview 2011–12.

[b5] Australian Institute of Health and Welfare and Melbourne/Australian Institute of Family Studies (AIHW/AIFS) (2012). Closing the Gap Clearinghouse: What works to overcome Indigenous disadvantage: key learnings and gaps in the evidence 2010–11.

[b6] Barry M, Wailes N (2004). Contrasting Systems? 100 Years of Arbitration in Australia and New Zealand. Journal of Industrial Relations.

[b7] Bessant J, Watts R, Dalton T, Smyth P (2006). Talking Policy: How social policy is made.

[b8] Boston J, Dalziel P, St John S (1999). Redesigning the Welfare State in New Zealand: Problems, Policies, Prospects.

[b9] Castles FG (1978). The Social Democratic Image of Society: A Study of the Achievement and Origins of Scandinavian Social Democracy in Comparative Perspective.

[b10] Castles FG (1985). The Working Class and Welfare: Reflections on the Political Development of the Welfare State in Australia and New Zealand, 1890–1980.

[b11] Castles FG (1988). Australian Public Policy and Economic Vulnerability: The Australian Experience.

[b12] Castles FG, Castles FG (1989). Social Protection by Other Means: Australia's Strategy of Coping With External Vulnerability. The Comparative History of Public Policy.

[b13] Castles FG (1992). On Sickness Days and Social Policy. Australian and New Zealand Journal of Sociology.

[b14] Castles FG (1994). The Wage Earners' Welfare State Revisited: Refurbishing the Established Model of Australian Social Protection, 1983–1993. Australian Journal of Social Issues.

[b15] Castles FG (1997a). Historical and comparative perspectives on the Australian welfare state: a response to Rob Watts. Journal of Sociology.

[b16] Castles FG (1997b). The institutional design of the Australian Welfare State. International Social Security Review.

[b17] Castles FG (2001). A Farewell to Australia's Welfare State. International Journal of Health Services.

[b18] Castles FG (2010). Black swans and elephants on the move: the impact of emergencies on the welfare state. Journal of European Social Policy.

[b19] Castles FG, Gerritsen R, Vowles J (1996). The Great Experiment: Labour Parties and Public Policy Transformation in Australia and New Zealand.

[b20] Castles FG, Mitchell D (1990). Three Worlds of Welfare Capitalism or Four?.

[b21] Castles FG, Mitchell D, Castles FG (1993). Worlds of Welfare and Families of Nations. Families of Nations: Patterns of Public Policy in Western Democracies.

[b22] Castles FG, Uhr J, Obinger H, Leibfried S, Castles FG (2005). Australia: Federal constraints and institutional innovations. Federalism and the Welfare State: New World and European Experiences.

[b23] Castles FG, Leibfried S, Lewis J, Obinger H, Pierson C (2012). The Oxford Handbook of the Welfare State.

[b24] Clark H (2002, 2008). Growing an Innovative New Zealand.

[b25] Australian Government (2008). Working Families Support Package.

[b26] Craig D, Bauld L, Clarke K, Maltby T (2006). Community well-being strategy and the legacies of new institutionalism and New Public Management in third way New Zealand. Social Policy Review 18: Analysis and debate in social policy, 2006.

[b27] Dalton T, McClelland A, Smyth P (2010). Housing policy in Australia: Big problems but well down the agenda. Social Policy in Australia: Understanding for Action.

[b28] Dean M (2012). Rethinking neoliberalism. Journal of Sociology.

[b29] Department of Labour (2012, 2011). Māori Labour Market Factsheet: March 2012.

[b30] Esping-Andersen G (1990). The Three Worlds of Welfare Capitalism.

[b31] Esping-Andersen G (1997). Hybrid or Unique?: the Japanese Welfare State Between Europe and America. Journal of European Social Policy.

[b32] Garton S, McCallum ME (1996). Workers' Welfare: Labour and the Welfare State in 20th-Century Australia and Canada. Labour History.

[b33] Gillard J (2010). Address to the nation. http://www.theaustralian.com.au/national-affairs/julia-gillards-address-to-the-nation/story-fn59niix-1225893284705.

[b34] Giovannini E (2008). Understanding economic statistics: an OECD perspective.

[b35] Harmer Review (2009). Pension Review Report.

[b36] Henry Review (2009, 2011). Australia's future tax system: Report to the Treasurer.

[b37] Herscovitch A, Stanton D (2008). History of social security in Australia. Family Matters.

[b38] HM Treasury (2012). Budget 2012.

[b39] HM Treasury (2013). Budget 2013.

[b40] Huber E, Stephens JD, Glyn A (2001). The Social Democratic Welfare State. Social Democracy in Neoliberal Times: The Left and Economic Policy since 1980.

[b41] Humpage L, Eversole R, McNeish J-A, Cimadamore AD (2005). Tackling Indigenous Disadvantage in the Twenty-First Century: ‘Social Inclusion’ and Maori in New Zealand. Indigenous Peoples and Poverty: An International Perspective.

[b42] Humpage L (2008). Radical Change or More of the Same?: Public Attitudes Towards Social Citizenship in New Zealand since Neoliberal Reform. Australian Journal of Social Issues.

[b43] Immervoll H (2010). Minimum-Income Benefits in OECD Countries: Policy Design, Effectiveness and Challenges.

[b44] International Monetary Fund (IMF) (2012). Fiscal Monitor: Balancing Fiscal Policy Risks.

[b45] Korpi W (1978). The Working Class in Welfare Capitalism: Work Unions and Politics in Sweden.

[b46] Korpi W, Palme J (1998). The Paradox of Redistribution and Strategies of Equality: Welfare State Institutions, Inequality, and Poverty in the Western Countries. American Sociological Review.

[b47] Lewis J, Plomien A (2009). ‘Flexicurity’ as a policy strategy: the implications for gender equality. Economy and Society.

[b48] Lunt N, O'Brien M, Stephens R (2008). New Welfare, New Zealand.

[b49] Mitchell D (2004). Reshaping Australian social policy: alternatives to the breadwinner welfare state.

[b50] Murphy J (2006). The other welfare state: Non-government agencies and the mixed economy of welfare in Australia. History Australia.

[b51] O'Brien M (2008, 2002). Poverty, policy and the state: The changing face of social security.

[b52] O'Connor JS, Orloff AS, Shaver S (1999). States, Markets, Families: Gender, Liberalism and Social Policy in Australia, Canada, Great Britain and the United States.

[b53] Organisation for Economic Cooperation and Development (OECD) (1972, 2012). Expenditure Trends in OECD Countries 1960–1980.

[b54] Organisation for Economic Cooperation and Development (OECD) (2007). Benefits and Wages 2007: OECD Indicators.

[b55] Organisation for Economic Cooperation and Development (OECD) (2008). Growing Unequal? Income Distribution and Poverty in OECD Countries.

[b56] Organisation for Economic Cooperation and Development (OECD) (2010). OECD Economic Surveys: Australia 2010.

[b57] Organisation for Economic Cooperation and Development (OECD) (2011a). Divided we stand: why inequality keeps rising.

[b58] Organisation for Economic Cooperation and Development (OECD) (2011b). http://www.oecd.org/els/oecdemploymentoutlook2011.htm.

[b59] Parker S (2013). The squeezed middle: The pressure on ordinary workers in America and Britain.

[b60] Quiggin J (1998). Social democracy and market reform in Australia and New Zealand. Oxford Review of Economic Policy.

[b61] Saunders P (1991). Selectivity and Targeting in Income Support: The Australian Experience. Journal of Social Policy.

[b62] Saunders P (1994). Welfare and Inequality: National and International Perspectives on the Australian Welfare State.

[b63] Saunders P (1999). Social Security in Australia and New Zealand: Means-tested or Just Mean?. Social Policy & Administration.

[b64] Saunders P (2011). Down and Out: Poverty and Exclusion in Australia.

[b65] Saunders P (2013). Reflections on the Concept of Social Exclusion and the Australian Social Inclusion Agenda. Social Policy & Administration.

[b66] Saunders P, Deeming C (2011). The Impact of the Crisis on Australian Social Security Policy in Historical Perspective. Social Policy & Administration.

[b67] Shalev M (2007). Limits and Alternatives to Multiple Regression in Comparative Research. Comparative Social Research.

[b68] Smyth P, Buchanan J (2013). Inclusive Growth in Australia: Social policy as economic investment.

[b69] Soldatic K, Pini B (2012). Continuity or Change? Disability Policy and the Rudd Government. Social Policy & Society.

[b70] Starke P (2008). Radical Welfare State Retrenchment a Comparative Analysis.

[b71] Stebbing A, Spies-Butcher B (2010). Universal Welfare by ‘Other Means’? Social Tax Expenditures and the Australian Dual Welfare State. Journal of Social Policy.

[b72] Stephens R, Boston J, Dalziel P, St John S (1999). Poverty, Family Finances and Social Security. Redesigning the Welfare State in New Zealand: Problems, Policies, Prospects.

[b73] Taylor-Gooby P (2013). The Double Crisis of the Welfare State and What We Can Do About It.

[b74] Tenbensel T, Mays N, Cumming J (2011). A successful mix of hierarchy and collaboration? Interpreting the 2001 reform of the governance of the New Zealand public health system. Policy & Politics.

[b75] Thomson D (1998). A World Without Welfare: New Zealand's Colonial Experiment.

[b76] Van Wanrooy B, Denemark D, Meagher G, Wilson S, Western M, Phillips T (2007). The quiet before the storm? Attitudes towards the new industrial relations system. Australian Social Attitudes 2.

[b77] Veit-Wilson J (1998). Setting adequacy standards: How governments define minimum incomes.

[b78] Watts R (1993). Australian Standards of Living: Some Gender Considerations. Australian Journal of Social Issues.

[b79] Watts R (1997). Ten years on: Francis G. Castles and the Australian ‘wage-earners’ welfare state. Journal of Sociology.

[b80] Castles FG (1998). The Really Big Trade-Off: Home Ownership and the Welfare State in the New World and the Old. Acta Politica.

[b81] Cheyne C, O′Brien M, Belgrave M Social Policy in Aotearoa New Zealand.

[b82] Christine K, Andrew JS, Melanie V (2009). A healthy diet consistent with Australian health recommendations is too expensive for welfare-dependent families. Australian and New Zealand Journal of Public Health.

[b83] Haworth N A Commentary on Politics and Employment Relations in New Zealand: 2008-2011. New Zealand Journal of Employment Relations.

[b84] Murphy L (2000). A profitable housing policy? The privatization of the New Zealand Government's residential mortgage portfolio. Regional Studies.

[b85] O'Sullivan J, Ashton T A Minimum Income for Healthy Living (MIHL) – Older New Zealanders. Ageing & Society.

[b86] Deeming C Determining Minimum Standards of Living and Household Budgets: Methodological Issues. Journal of Sociology.

[b87] Nolan P

[b88] Stephens R (2000). The Social Impact of Reform: Poverty in Aotearoa/New Zealand. Social Policy & Administration.

